# Meek Micrografting Technique for Reconstruction of Extensive Necrotizing Fasciitis of the Anterior Abdomen and Bilateral Femoral Region: A Case Report

**DOI:** 10.1055/a-2077-5745

**Published:** 2023-08-24

**Authors:** Jyi Cheng Ng, Ahmad Ibrahim Ahmad Zaidi, Jun De Lee, Mohd Faisal Jabar

**Affiliations:** 1 Department of Surgery, Faculty of Medicine and Health Sciences, Universiti Putra Malaysia, Serdang, Malaysia; 2 Department of Surgery, Universiti Putra Malaysia Teaching Hospital, Serdang, Malaysia

**Keywords:** necrotizing fasciitis, reconstructive surgery, skin transplantation, skin graft

## Abstract

Necrotizing fasciitis is an uncommon yet fatal soft tissue infection. Current recommended treatment includes antibiotics with repeat surgical exploration and wound debridement followed by reconstruction. In burn patients, the Meek micrograft has demonstrated a higher true expansion ratio, faster reepithelialization rate, more resilient toward infection, and reduced risk of graft failure as compared with meshed graft. To our best knowledge, the use of Meek micrografting technique in reconstruction of postdebridement wounds of necrotizing fasciitis has not been reported. Hereby, we present a case of a 57-year-old gentleman who was referred to us for wound reconstruction after surgical debridement of Fournier's gangrene and extensive necrotizing fasciitis involving the anterior abdomen and bilateral femoral region. Meek micrografting technique was used to reconstruct the anterior abdomen as the wound bed was large. Although the graft was complicated with a small area of localized infection, it did not spread across the entire graft and was successfully treated with topical antibiotics and regular wound dressing. In our case, wound reconstruction using Meek micrografting technique in a patient with extensive necrotizing fasciitis was successful and showed positive outcome. Therefore, we suggest further studies to be conducted to investigate the applications and outcomes of the Meek micrografting technique, especially in patients with extensive wound bed and limited donor site availability.

## Introduction

Fournier's gangrene is defined as an infective necrotizing fasciitis of the perineal, genital, or perianal region. 1 The infection usually extends following the course of the superficial perineal fascia that is in continuity with Colles fascia and Scarpa's fascia, into the abdominal wall as well as the legs. Necrotizing fasciitis carries a high morbidity and mortality rate, especially when intervention is delayed as the infection spreads rapidly. 2 Urgent and radical debridement of devitalized tissue is crucial in ceasing the progression of the infection. Meshed split-thickness skin graft (SSG) is usually used thereafter for reconstruction, but it has a limited expansion ratio leading to increased donor site morbidity when grafted on extensive wounds. 3

First introduced by Cicero Parker Meek in 1958 for the use in burn patients, 4 Meek micrograft technique was quickly overshadowed by the mesh technique due to cheaper and faster production of meshed grafts. 5 In the 1990s, this technique was revived by physicians in Beverwijk and later modified by Kreis and Raff to take advantage of its higher expansion ratio over mesh grafts. 5 The use of the Meek micrograft technique in burn patients has been widely discussed. It has shown to be a viable alternative for SSG in burns patients, due to a higher expansion ratio, faster reepithelialization rate, can be used in wounds with poor vascularity, and is also superior in terms of long-term scar and functional outcome. 3 6 7 8 It is especially favorable in burn patients with limited autograft donor sites. 6 Here, we describe a case of successful reconstruction using the Meek micrografting technique in a patient with a large anterior abdomen wound bed postdebridement for extensive necrotizing fasciitis. The patient provided written informed consent for the publication and use of his images.

## Case

**Fig. 1:**
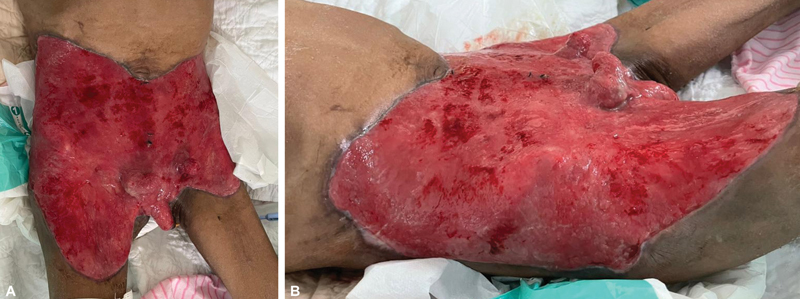
( **A** ) Granulation tissue seen over the anterior abdomen and bilateral femoral region extending to the proximal thigh after three surgical exploration and wound debridement (anterior view). ( **B** ) Granulation tissue seen over the anterior abdomen and bilateral femoral region extending to the proximal thigh after three surgical explorations and wound debridement (lateral view).

A 57-year-old gentleman with underlying hypertension and meatal stenosis was referred to us for wound reconstruction postsurgical debridement for Fournier's gangrene and extensive necrotizing fasciitis involving the anterior abdomen and bilateral femoral region. In addition, he also developed obstructive uropathy and bladder stone. He has completed surgical exploration and wound debridement over the penis, scrotum, lower abdomen, and bilateral anterior femoral region thrice: on day 1, 5, and 10, respectively ( Fig. 1A and B ). The debridement was comanaged by the surgical, orthopaedics, and urological team. Intraoperative findings revealed necrotic tissue involving the anterior abdomen, suprapubic, paraumbilical, and bilateral flank region, extending down to the bilateral femoral region, with Fournier's gangrene. After multiple wound debridements, the patient was not in good condition with prolonged hospitalization, in which he developed septic shock, bacteremia with deep wound infection, evidenced by methicillin-resistant *Staphylococcus aureus* and *Pseudomonas aeruginosa* cultured on deep tissue culture and sensitivity, despite meticulous efforts to reduce the risk of infection, such as granulation tissue debridement and thorough irrigation with hydrogen peroxide, povidone–iodine, and copious saline prior to tissue sampling for culture and sensitivity.

**Fig. 2:**
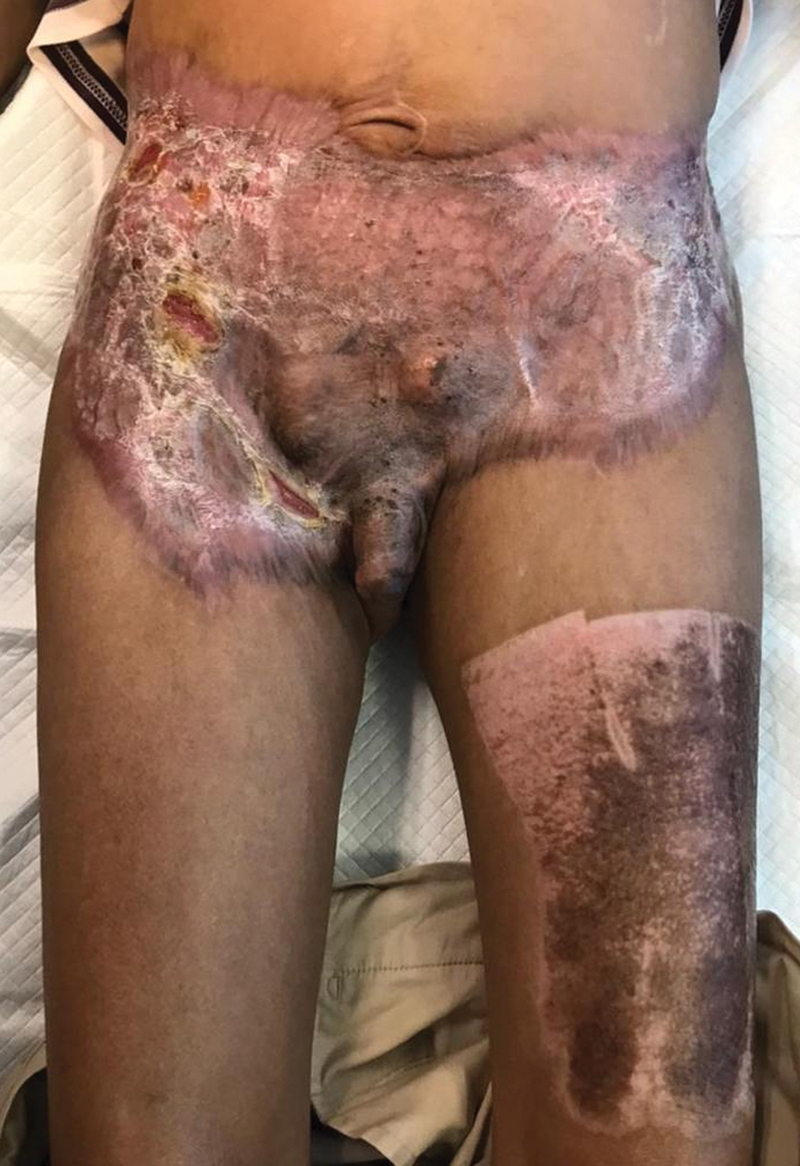
The donor site is on the left anterior thigh, where split-thickness skin graft was harvested. The donor site is relatively small in comparison to the graft site. The picture was taken 6 weeks after the operation.

**Fig. 3:**
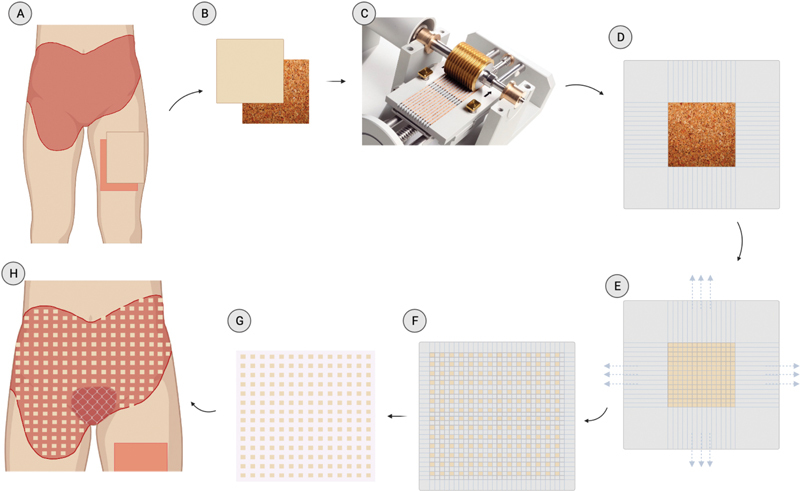
Meek micrograft technique. ( **A** ) Split-thickness skin graft (SSG) was harvested from the left anterior thigh. ( **B** ) SSG was fitted in a 42 mm × 42 mm damp cork base with the dermal side inward. ( **C** ) SSG was placed into the Meek Mesher (Humeca) along with cork squares to be cut into 14 × 14 small squares (total of 196 small “postage stamp” squares, 3 mm × 3 mm each). ( **D** ) An adhesive dressing spray was sprayed on the epidermal side of the grafts, and it was pressed onto a prefolded polyamide gauze with aluminum backing with an expansion ratio of 1:6. ( **E** ) The cork was then removed, leaving the graft on the prefolded polyamide gauze. ( **F** ) The pleated sheath was extended at all sides until it became entirely unfolded. ( **G** ) The aluminum backing was removed, leaving the polyamide gauze with the expanded autografts ready for grafting. ( **H** ) The margins were trimmed, and the gauze was applied to the wound bed and secured with surgical staples.

**Fig. 4:**
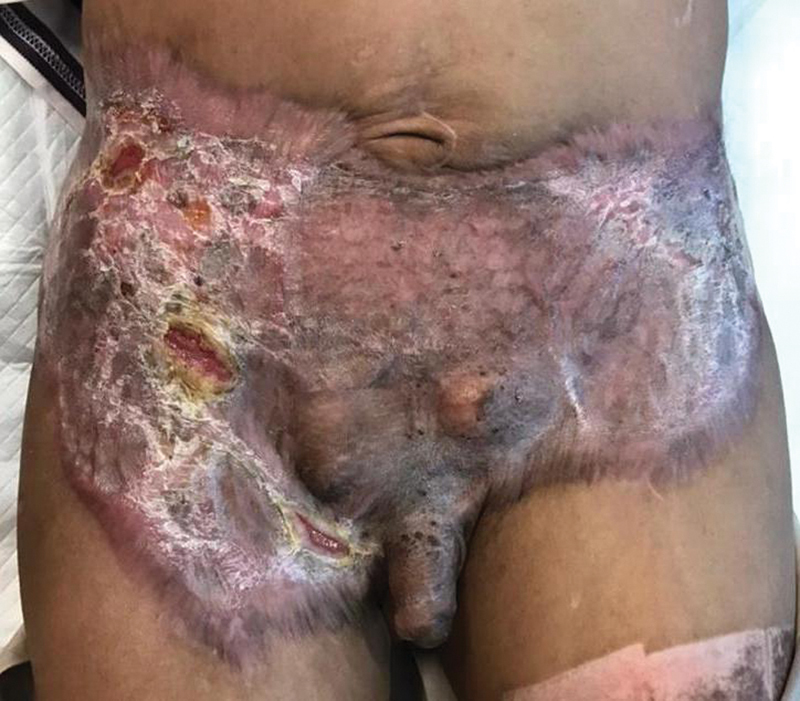
Graft complicated with infection, localized to a small area over the right flank, right iliac fossa, and right inguinal region, sparing the central and left parts of the graft. The picture was taken 2 months after the operation.

**Fig. 5:**
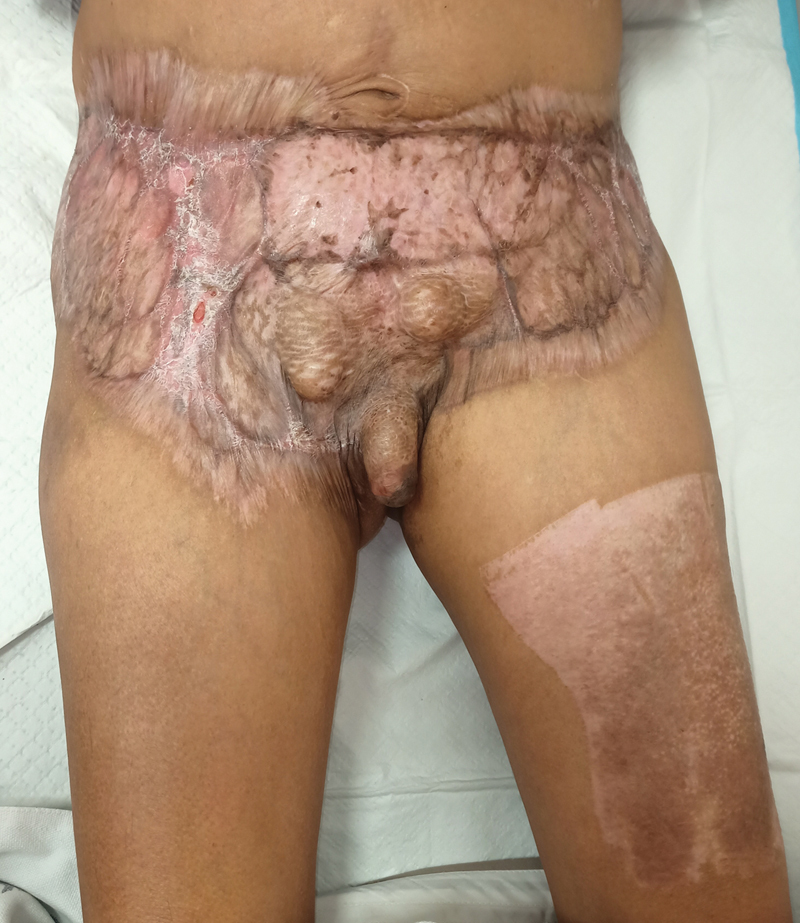
The wound bed and donor site healed well with minimal contracture. The picture was taken 1 year after the operation.

Unfortunately, wound reconstruction could not be done earlier due to complications of the infection and there were no plastic surgeons in the primary care team hospital. In addition, our patient was facing financial difficulties and the primary team could not refer him to other institutions. Therefore, our team was invited to the hospital to perform the case, and wound reconstruction was done on day 65 since the first debridement. The testes were placed in the lower abdomen by the primary team following previous surgical debridements ( Fig. 1A and B ). The testes were plastered to the wound bed, and there is a high risk of testicular artery bleeding if we attempt to separate the testes from the abdomen. After discussing the risk with our patient, abdominal implantation of the testicles was done instead of scrotal reconstruction. SSG was harvested from the left anterior thigh ( Fig. 2 ). Part of the SSG was meshed with an expansion ratio of 1:3 and grafted over the testes. The reconstruction of the circumferential penile shaft was done by meshing the SSG without expanding it. As the involvement of the anterior abdomen was extensive ( Fig. 1A and B ), with an estimated total body surface area of 10%, we decided to use the Meek micrografting technique ( Fig. 3 ) with an expansion ratio of 1:6. It was done by cutting the SSG to be fitted in a 42 mm × 42 mm damp cork base with the dermal side downward. The autografts along with cork squares were placed into the Meek Mesher (Humeca) where the autografts were cut into 14 × 14 small squares, with a total of 196 small “postage stamp” squares measuring 3 mm × 3 mm each. Next, an adhesive dressing spray was sprayed on the epidermal side of the grafts, and it was pressed onto a prefolded polyamide gauze with aluminum backing with an expansion ratio of 1:6. The cork was then removed, and the pleated sheath was extended at all sides until it became entirely unfolded. The aluminum backing was removed, leaving the polyamide gauze with the expanded autografts ready for grafting. The margins were trimmed and the gauze was applied to the wound bed and secured with surgical staples. The donor site and graft sites were properly managed with appropriate dressings. Preemptive scar massage and pressure garment were applied over the grafted and donor site to prevent contracture. Fortunately, our patient survived after 3 months of intensive inpatient care with resolved candidemia and resolved multiresistant *Klebsiella pneumoniae* bacteremia. He was discharged upon request 3 weeks after the reconstructive surgery. During follow-up, we noticed a small area of infection over the graft on the anterior abdomen ( Fig. 4 ). The infection was localized and did not spread across the entire graft. He was treated as an outpatient with topical hydrocortisone and neomycin with regular wound dressing. The infection was well-controlled and healed without complications ( Fig. 5 ).

## Discussion

About one in five patients with necrotizing fasciitis died, 9 10 11 and the mortality rate is even higher when intervention is delayed. 10 11 Necrotizing fasciitis is a fatal, aggressive, and rapidly progressive disease. It is a clinical diagnosis, and the cornerstone of management of this lethal condition is to reduce systemic toxicity, halt the progression of the infection, and eliminate the causative microorganisms, achieved via urgent hemodynamic resuscitation, broad-spectrum antibiotics and repeat surgical exploration, and wound debridement, 12 13 followed by skin grafting and complex genital reconstructive surgery. 13 Early and extensive debridement of necrotic and nonviable tissues together with a slim window of healthy adjacent tissue is crucial in ceasing the progression of the devastating infection and reducing mortality. 10 11 12 The procedure may be repeated to achieve adequate infection control. A previous study has suggested a mean of 3.5 debridement operations per patient, 14 which is in keeping with our patient who completed surgical debridement thrice.

After multiple wound debridement, our patient was not in good condition with prolonged hospitalization as he was unable to sit up or ambulate. He also developed bacteremia with deep wound infection, despite meticulous efforts to reduce the risk of infection, such as granulation tissue debridement and thorough washing with hydrogen peroxide, povidone–iodine, and copious saline prior to tissue sampling for culture and sensitivity. In view of the patient's condition, we decided to opt for Meek micrograft because it has a better graft resilience toward infection, and a higher true expansion ratio to cover the extensive wound bed, along with minimal donor site morbidity and reduced risk of graft failure. 3 6 7 8 This is as opposed to meshed SSG where there will be a higher graft load, increased risk of graft infection and failure, as well as prolonged hospitalization. 3 7 8

Meshed SSG has a limited true expansion ratio of 1:1.5, which exhibits a significant discrepancy from the theoretical expansion ratio. 3 Even though an expansion ratio of up to 1:6 has been reported with skin meshers, the majority did not reach the claimed expansion when the ratio becomes greater than 1:3 mesher. 15 When it is further expanded to a larger ratio, it leaves significant gaps in between, exposing a larger area of wound bed that may potentially impede reepithelialization or even graft failure, in addition to the higher risk of infection. 8 In contrast, Meek micrograft has a higher true expansion ratio of 1:3 or up to 1:9, 3 without compromising the reepithelialization rate and risk of extensive infection. 8 16 The high expansion ratio is particularly important in our patient as we were able to reconstruct an extensive area without increasing donor site morbidity.

Infection is a significant complication following skin allograft transplantation, as it often results in graft loss. 17 18 Meek grafts have been reported to be more resilient toward infection when compared with meshed SSG. 8 19 This is an unexpected observation seen in our patient, who developed an infection over the graft at the right anterior abdomen. Surprisingly, the infection was contained and did not spread across the entire graft. There was no graft failure necessitating regrafting. A proposed explanation is that the skin autograft islands in Meek technique lack continuity between each other, limiting the progression of infection across the wound bed. This is unlike meshed SSG, which often results in the loss of the whole graft, as infection could creep under the entire graft causing loss of contact. 8 Our patient was treated successfully with topical antibiotics and regular wound dressing without regrafting. This is consistent with a study by Medina et al, where the Meek skin grafts survived and resumed the reepithelialization process in patients with graft infection treated with topical management and systemic antibiotic therapies. 19

As for penile reconstruction, we decided to use the mesh graft instead of the unfenestrated sheet graft to allow better drainage of hematomas or seromas. 20 A randomized trial has shown a lower percentage of graft loss due to hematoma formation in meshed grafts as compared with sheet grafts. 20 Even though the graft was meshed, we did not expand the graft to reduce contracture. There was minimal contracture seen in our patient postreconstruction, as we prevented it early with meticulous technique, in which preemptive scar massage and pressure garment were applied over the grafted and donor site.

Although tedious and laborious, Meek micrografting technique is a feasible option for the reconstruction of postdebridement wounds in patients with extensive necrotizing fasciitis. There is a paucity of data reporting the outcome of Meek micrograft technique other than in burn patients. Therefore, we suggest further studies to be conducted to investigate the applications and outcomes of the Meek Micrografting technique, especially in patients with extensive wound beds and limited donor site availability.

## References

[1] Smith G L Bunker C B Dinneen M D Fournier's gangrene Br J Urol 1998 81 03 347 355 9523650 10.1046/j.1464-410x.1998.00532.x

[2] Radcliffe R S Khan M A Mortality associated with Fournier's gangrene remains unchanged over 25 years BJU Int 2020 125 04 610 616 31975540 10.1111/bju.14998

[3] Quintero E C Machado J FE Robles R AD Meek micrografting history, indications, technique, physiology and experience: a review article J Wound Care 2018 27(Sup2):S12 S18 10.12968/jowc.2018.27.Sup2.S12 29419365

[4] Meek C P Successful microdermagrafting using the Meek-Wall microdermatome Am J Surg 1958 96 04 557 558 13571547 10.1016/0002-9610(58)90975-9

[5] Ottomann C Hartmann B Branski L Krohn C A tribute to Cicero Parker Meek Burns 2015 41 08 1660 1663 26233898 10.1016/j.burns.2015.06.013

[6] Houschyar K S Tapking C Nietzschmann I Five years experience with Meek grafting in the management of extensive burns in an adult burn center Plast Surg (Oakv) 2019 27 01 44 48 30854361 10.1177/2292550318800331 PMC6399769

[7] Lee S Z Halim A S Superior long term functional and scar outcome of Meek micrografting compared to conventional split thickness skin grafting in the management of burns Burns 2019 45 06 1386 1400 31054957 10.1016/j.burns.2019.04.011

[8] Noureldin M A Said T A Makeen K Kadry H M Comparative study between skin micrografting (Meek technique) and meshed skin grafts in paediatric burns Burns 2022 48 07 1632 1644 35248428 10.1016/j.burns.2022.01.016

[9] Dhanasekara C S Marschke B Morris E Kahathuduwa C N Dissanaike S Regional variations in microbiology and outcomes of necrotizing soft tissue infections: a systematic review and meta-analysis Surg Infect (Larchmt) 2022 23 07 634 644 35904966 10.1089/sur.2022.142

[10] Gelbard R B Ferrada P Yeh D D Optimal timing of initial debridement for necrotizing soft tissue infection: a practice management guideline from the Eastern Association for the Surgery of Trauma J Trauma Acute Care Surg 2018 85 01 208 214 29485428 10.1097/TA.0000000000001857

[11] Nawijn F Smeeing D PJ Houwert R M Leenen L PH Hietbrink F Time is of the essence when treating necrotizing soft tissue infections: a systematic review and meta-analysis World J Emerg Surg 2020 15 4 31921330 10.1186/s13017-019-0286-6 PMC6950871

[12] Singh A Ahmed K Aydin A Khan M S Dasgupta P Fournier's gangrene. A clinical review Arch Ital Urol Androl 2016 88 03 157 164 27711086 10.4081/aiua.2016.3.157

[13] Tarasconi A Perrone G Davies J Anorectal emergencies: WSES-AAST guidelines World J Emerg Surg 2021 16 01 48 34530908 10.1186/s13017-021-00384-x PMC8447593

[14] Chawla S N Gallop C Mydlo J H Fournier's gangrene: an analysis of repeated surgical debridement Eur Urol 2003 43 05 572 575 12706005 10.1016/s0302-2838(03)00102-7

[15] Kamolz L P Schintler M Parvizi D Selig H Lumenta D B The real expansion rate of meshers and micrografts: things we should keep in mind Ann Burns Fire Disasters 2013 26 01 26 29 23966895 PMC3741003

[16] Dahmardehei M Vaghardoost R Saboury M Comparison of modified Meek technique with standard mesh method in patients with third degree burns World J Plast Surg 2020 9 03 267 273 33330002 10.29252/wjps.9.3.267 PMC7734932

[17] Unal S Ersoz G Demirkan F Arslan E Tütüncü N Sari A Analysis of skin-graft loss due to infection: infection-related graft loss Ann Plast Surg 2005 55 01 102 106 15985801 10.1097/01.sap.0000164531.23770.60

[18] Rode H Martinez R Potgieter D Adams S Rogers A D Experience and outcomes of micrografting for major paediatric burns Burns 2017 43 05 1103 1110 28318749 10.1016/j.burns.2017.02.008

[19] Medina A Riegel T Nystad D Tredget E E Modified Meek micrografting technique for wound coverage in extensive burn injuries J Burn Care Res 2016 37 05 305 313 27355651 10.1097/BCR.0000000000000244

[20] Nikkhah D Booth S Tay S Gilbert P Dheansa B Comparing outcomes of sheet grafting with 1:1 mesh grafting in patients with thermal burns: a randomized trial Burns 2015 41 02 257 264 25175303 10.1016/j.burns.2014.07.023

